# Hydatid Pulmonary Embolism Complicating Hepatopulmonary Hydatid Disease: A Case Report

**DOI:** 10.7759/cureus.112716

**Published:** 2026-07-15

**Authors:** Leila Salak, Soumia Fdil, Dalal Zagaouch, Khalid Bouti, Sanaa Hammi

**Affiliations:** 1 Department of Pulmonology, Mohammed VI University Hospital, Tangier, MAR; 2 Faculty of Medicine and Pharmacy of Tangier, Abdelmalek Essaâdi University, Tangier, MAR

**Keywords:** echinococcus granulosus, hydatid disease, hydatid pulmonary embolism, pulmonary hypertension, thoracic ct angiography

## Abstract

Hydatid disease is an endemic parasitic infection that primarily affects the liver and lungs. Hydatid pulmonary embolism is an exceptional and potentially severe complication, usually resulting from the rupture of a hydatid cyst into the venous circulation. We report the case of a 64-year-old patient, a former smoker from a rural area, who had been diagnosed with hepatopulmonary hydatid disease complicated by chronic bilateral hydatid pulmonary embolism two years before the current admission. Echinococcosis serology was positive. Thoracic computed tomography (CT) angiography revealed arterial hydatid involvement associated with multiple pulmonary and hepatic cystic lesions. Management included therapeutic anticoagulation, empirical antibiotics, and the cautious reintroduction of albendazole with close biological monitoring, followed by hepatic surgery.

Pulmonary embolism is a rare presentation of hydatid disease. Because of its nonspecific clinical features, careful diagnostic assessment and individualized management are required.

## Introduction

Hydatid disease is a parasitic disease caused by *Echinococcus granulosus* [1]. It preferentially affects the liver and lungs [2,3]. It is common in Mediterranean countries [4]. Hydatid pulmonary embolism is a rare and serious complication of echinococcosis. It occurs when a hydatid cyst ruptures into the venous return system, often from hepatic origin [5]. Diagnosis relies primarily on imaging, particularly computed tomography (CT) and magnetic resonance imaging (MRI). The management of hydatid pulmonary embolism should be individualized according to the extent of vascular involvement, associated hydatid lesions, complications, and the patient's surgical risk. Surgery remains the preferred treatment when feasible, whereas medical therapy with albendazole may be used as an adjunct to surgery or as the main treatment in chronic emboli and diffuse pulmonary arterial involvement or when surgery is contraindicated [6]. We report a rare case of chronic hydatid pulmonary embolism complicating hepatopulmonary hydatid disease, highlighting the associated diagnostic and therapeutic challenges and the need for individualized management in complex clinical settings.

## Case presentation

We report the case of a 64-year-old patient, a former smoker, from a rural area, with a history of close exposure to livestock and dogs in childhood. He had been diagnosed with hepatopulmonary hydatid disease two years before the current admission. Albendazole therapy was initiated but subsequently discontinued after six weeks because of significant liver cytolysis. Surgery was not initially indicated due to the high operative risk and the complexity of hepatopulmonary and vascular involvement.

In February 2026, the patient was admitted to our department for progressively worsening dyspnea, reaching modified Medical Research Council (mMRC) grade 2, with an intermittent cough, occurring in a context of general deterioration. On admission, the clinical examination revealed a conscious patient, hemodynamically and respiratory stable, with an oxygen saturation of 98% on room air, a blood pressure of 120/60 mmHg, a heart rate of 80 beats per minute, and a respiratory rate of 21 breaths per minute. The pleuropulmonary and abdominal examination was unremarkable.

The biological tests revealed an inflammatory syndrome and elevated C-reactive protein levels. Hydatid serology was positive, and D-dimer levels were elevated. The biological test results are summarized in Table 1.

**Table 1:** Biological test results The values shown in bold indicate abnormal laboratory results outside the reference range

Parameter	Result	Reference Values
Leukocytes	16.6 × 10⁹/L	4-10 × 10⁹/L
Neutrophils	13.2 × 10⁹/L	1.5-7 × 10⁹/L
Lymphocytes	1.78 × 10⁹/L	1-4 × 10⁹/L
Eosinophils	0.6 × 10⁹/L	0.1-0.4 × 10⁹/L
Hemoglobin (HGB)	13.0 g/dL	13-16 g/dL
Platelets (PLTs)	243 × 10⁹/L	150-450 × 10⁹/L
C-reactive protein (CRP)	300 mg/L	<5 mg/L
Hydatid serology	Positive (>1/320)	<1/160, nonsignificant; 1/160, doubtful; >1/160, significant reaction suggestive of active hydatid disease
D-dimer	2,000 ng/mL	<500 ng/mL

**Figure 1:**
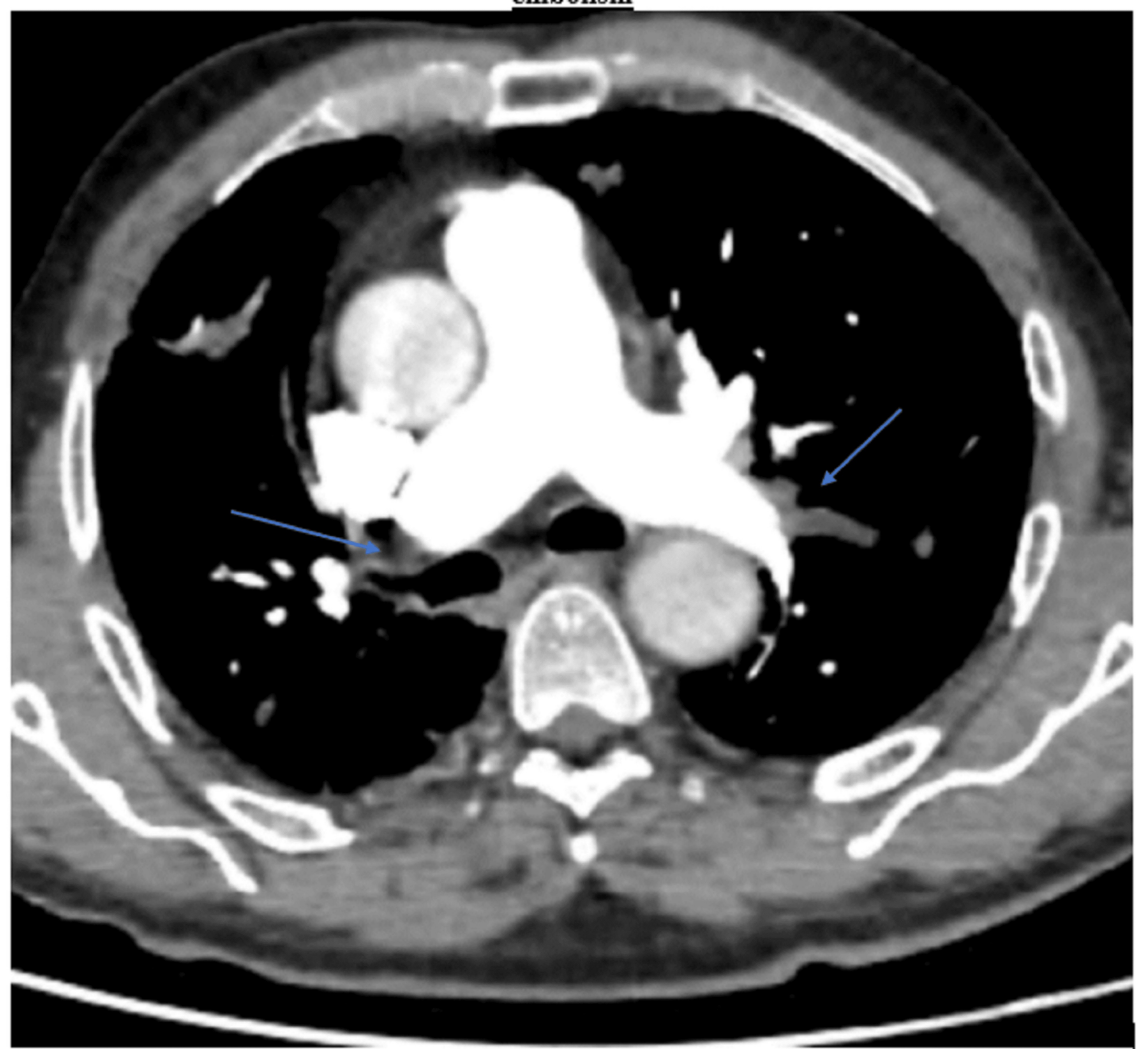
Axial contrast-enhanced chest CT showing bilateral hydatid pulmonary embolism CT: computed tomography

**Figure 2:**
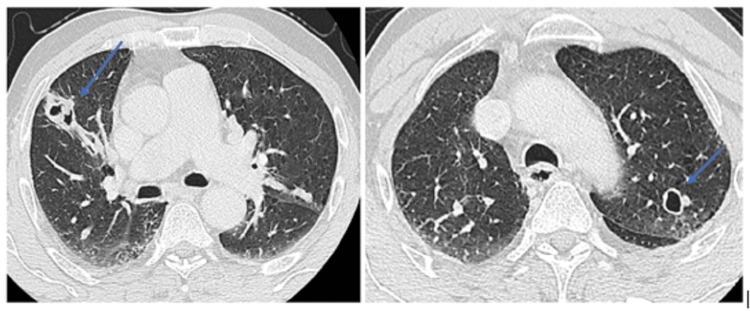
Axial thoracic CT scan showing bilateral pulmonary hydatid cysts with rupture and bronchial fistulization of the right hydatid cyst CT: computed tomography

**Figure 3:**
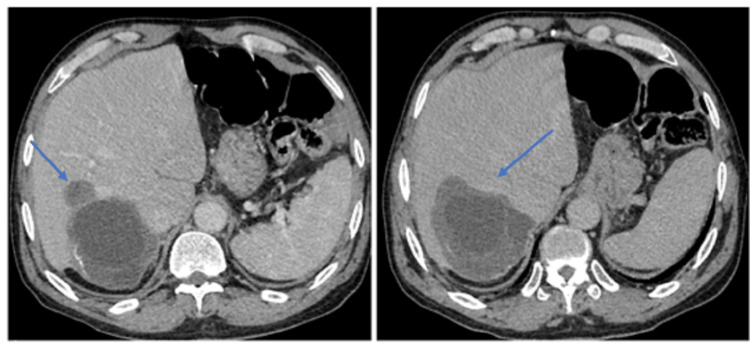
Axial abdominal CT showing hepatic hydatid cyst CT: computed tomography

The initial thoracoabdominal CT scan, performed at the time of diagnosis two years before the current admission, revealed proximal pulmonary embolism involving the right middle and lower lobes and distal embolism of the left upper lobe, associated with multiple ruptured pulmonary hydatid cysts in the right hemithorax with right bronchial fistulization. A ruptured cavitary hydatid cyst was also present in the left lung. A hepatic hydatid cyst classified as WHO-Informal Working Group on Echinococcosis (IWGE) Cystic Echinococcosis 2 (CE2), fissured at the basithoracic level, was also present. Two years after the initial diagnosis, because of progressive worsening of dyspnea, a follow-up thoracic CT angiography showed a slight increase in peripheral hydatid vascular involvement in the left upper lobe, with a cavitary apical lesion and regression in size of the hepatic lesion (Figures 1-3).

**Figure 4:**
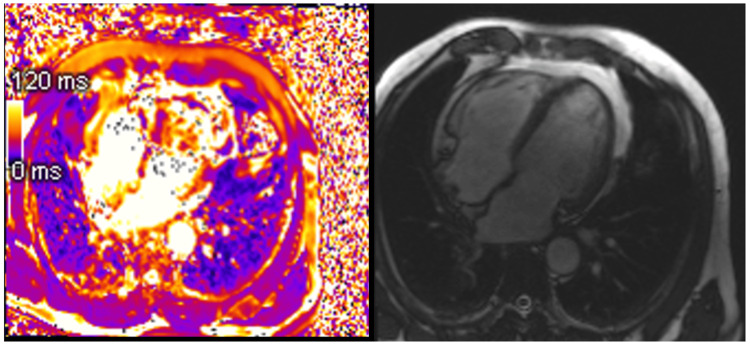
Cardiac MRI showing no evidence of cardiac hydatid involvement MRI: magnetic resonance imaging

Cardiac MRI showed no evidence of cardiac hydatid involvement, with preserved biventricular systolic function, a left ventricular ejection fraction (LVEF) of 60%, and no late gadolinium enhancement (Figure 4). Subsequent transthoracic echocardiography revealed preserved left ventricular systolic function, borderline right ventricular dilatation with preserved function, and a high probability of pulmonary hypertension, with a tricuspid regurgitation velocity of 3.5 m/second and an estimated systolic pulmonary artery pressure of 59 mmHg.

**Figure 5:**
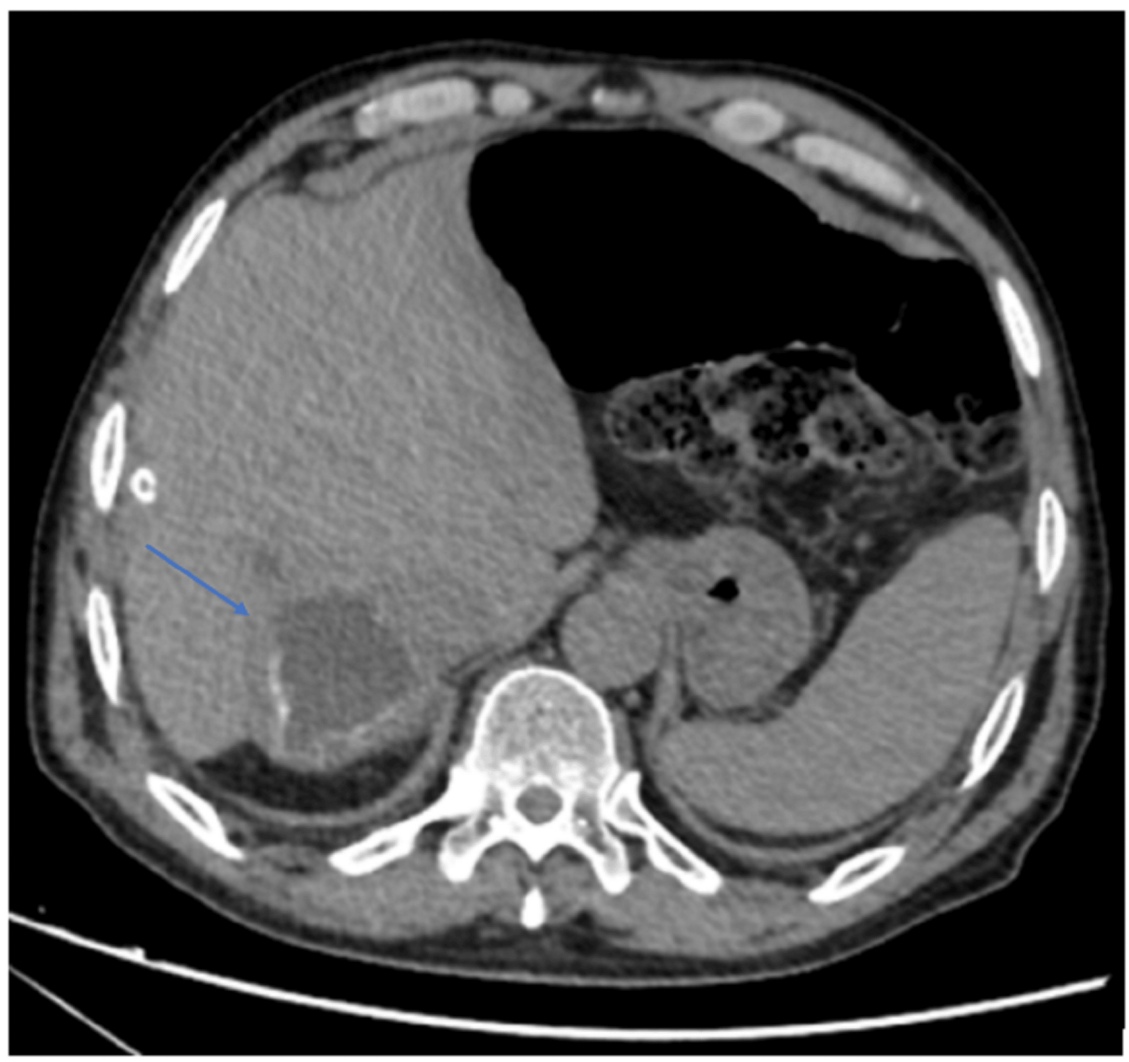
One-year follow-up abdominal CT scan CT: computed tomography

Therapeutic anticoagulation was initiated because a concomitant thrombotic component could not be definitively excluded, particularly in the context of elevated D-dimer levels and chronic pulmonary arterial involvement. Anticoagulation was therefore intended to target a possible superimposed thrombotic component, rather than the hydatid embolic material itself, for which no established therapeutic benefit has been demonstrated. Empirical antibiotic therapy was also initiated for the associated infectious syndrome. After the improvement of liver function tests and clinical stabilization, albendazole was cautiously reintroduced after multidisciplinary discussion, with close biological monitoring. The patient had a favorable clinical and biological outcome, with the improvement of dyspnea and the regression of the inflammatory syndrome. Follow-up CT imaging also showed a decrease in the size of the hepatic hydatid cyst (Figure 5). Hepatic surgery was subsequently performed, consisting of the resection of the protruding dome, lavage, and drainage. The postoperative course was favorable, with no immediate complications. The patient was reassessed 15 days after hospital discharge and subsequently at monthly intervals over a five-month follow-up period. Clinical examination and liver function testing were performed at each visit, with radiological and echocardiographic reassessment during follow-up. Albendazole remained well-tolerated, and the patient remained clinically stable under multidisciplinary surveillance.

## Discussion

Hydatid disease remains a public health problem, particularly in endemic areas [6,7]. Hydatid cysts develop primarily in the liver (65%) and lungs (25%). Cardiovascular locations are rare (0.5%-2%) [8]. Hydatid embolism is a non-thrombotic pulmonary embolism with a low risk of morbidity but a high risk of mortality [9]. Hydatid embolism is secondary to the rupture of a hydatid cyst located in the right side of the heart or, more rarely, to the rupture of a hepatic cyst in the inferior vena cava or the hepatic veins [10]. A more exceptional pathway has also been reported in the literature, corresponding to the direct extension of a pulmonary hydatid cyst toward the pulmonary artery [3,4]. Hydatid pulmonary embolism results from the migration of hydatid cyst material into the pulmonary circulation, rather than thrombotic material. In contrast, the existence of a true thrombophilic potential associated with hydatid disease remains uncertain, although rare cases of concomitant venous thrombosis have been reported [9,11].

The clinical presentation is nonspecific, ranging from simple malaise associated with a skin rash to anaphylactic shock, particularly in the acute phase. It can also manifest with clinical signs similar to those observed in thrombotic pulmonary embolism, including dyspnea, cough, hemoptysis, and chest pain. In some cases, the diagnosis may be made incidentally during the staging workup for hydatid disease [4,11-13]. The diagnosis is primarily based on imaging. Thoracic CT angiography plays a crucial role in confirming the diagnosis; it demonstrates well-circumscribed intra-arterial endoluminal defects of fluid attenuation with well-defined borders, with peripheral wall enhancement after contrast administration. It may also reveal associated findings, including vascular ectasia, thrombosis, or dilatation [6,14,15]. Compared with CT, MRI allows better characterization of the cystic nature of the lesion [16]. Transthoracic echocardiography remains the main diagnostic tool in cardiac hydatid disease, allowing the assessment of the hemodynamic repercussions of the embolism [3,17].

No standardized treatment guidelines have been established for hydatid pulmonary embolism. Although medical treatment with albendazole and mebendazole has shown promising results and is also the only therapeutic option for inoperable patients [11,16,18]. Surgery remains the first-line treatment when the patient's clinical condition allows. The surgical procedure involves two steps: the removal of the intra-arterial cysts, followed by the surgical management of the affected lung, either by a conservative approach or by total pneumonectomy [5,11,16]. The role of anticoagulation remains poorly defined and should not be considered an established treatment for parasitic embolic material but may be considered in selected cases when concomitant venous thrombosis or a superimposed thrombotic component is suspected [4,6,19].

## Conclusions

Hydatid pulmonary embolism is a very rare but potentially life-threatening complication of hydatid disease that poses significant diagnostic and therapeutic challenges. This case underscores the importance of integrating imaging findings with individualized therapeutic decision-making to optimize outcomes in patients with complex hepatopulmonary hydatid disease.
